# The association between objective walkability, neighborhood socio-economic status, and physical activity in Belgian children

**DOI:** 10.1186/s12966-014-0104-1

**Published:** 2014-08-23

**Authors:** Sara D’Haese, Delfien Van Dyck, Ilse De Bourdeaudhuij, Benedicte Deforche, Greet Cardon

**Affiliations:** Faculty of Medicine and Health Sciences, Department of Movement and Sports Sciences, Ghent University, Watersportlaan 2, Ghent, 9000 Belgium; Research Foundation Flanders (FWO), Egmontstraat 5, Brussels, 1000 Belgium; Department of Human Biometrics and Biomechanics, Vrije Universiteit Brussel, Brussels, Belgium

**Keywords:** GIS, Exercise, Physical environment, Youth, Walking, Cycling, Neighborhood, Children, Activity

## Abstract

**Background:**

Objective walkability is an important correlate of adults’ physical activity. Studies investigating the relation between walkability and children’s physical activity are scarce. However, in order to develop effective environmental interventions, a profound investigation of this relation is needed in all age groups. The aim of this study was to investigate the association between objective walkability and different domains of children’s physical activity, and to investigate the moderating effect of neighborhood socio-economic status in this relation.

**Methods:**

Data were collected between December 2011 and May 2013 as part of the Belgian Environmental Physical Activity Study in children. Children (9–12 years old; n = 606) were recruited from 18 elementary schools in Ghent (Belgium). Children together with one of their parents completed the Flemish Physical Activity Questionnaire and wore an accelerometer for 7 consecutive days. Children’s neighborhood walkability was calculated using geographical information systems. Multilevel cross-classified modeling was used to determine the relationship between children’s PA and objectively measured walkability and the moderating effect of neighborhood SES in this relation.

**Results:**

In low SES neighborhoods walkability was positively related to walking for transportation during leisure time (β = 0.381 ± 0.124; 95% CI = 0.138, 0.624) and was negatively related to sports during leisure time (β = −0.245 ± 0.121; 95% CI = −0.482, −0.008). In high socio-economic status neighborhoods, walkability was unrelated to children’s physical activity. No relations of neighborhood walkability and neighborhood socio-economic status with cycling during leisure time, active commuting to school and objectively measured moderate- to vigorous-intensity physical activity were found.

**Conclusions:**

No univocal relation between neighborhood walkability and physical activity was found in 9–12 year old children. Results from international adult studies cannot be generalized to children. There is a need in future research to determine the key environmental correlates of children’s physical activity.

**Electronic supplementary material:**

The online version of this article (doi:10.1186/s12966-014-0104-1) contains supplementary material, which is available to authorized users.

## Background

Although physical activity (PA) is associated with health benefits [1], many children do not meet the PA guidelines of 60 minutes of daily moderate- to vigorous-intensity physical activity (MVPA) [2]. In order to develop effective interventions to promote children’s PA, insight into potential determinants of PA is necessary [3].

Ecological models state that besides individual factors, environmental factors (e.g. physical environment) can influence PA behaviors [4] directly as well as indirectly [5]. These theories suggest a profound investigation of the neighborhood environment in order to create suitable interventions.

Walkability is a thoroughly studied physical environmental factor in relation to adults’ PA [6]. A high walkable neighborhood is characterized by high residential density, high street connectivity and high land use mix diversity [7]. In studies from the USA, Australia and Europe, higher objectively measured walkability has been consistently related to higher PA levels and more active transportation in adults [8-11]. It is important to find out if walkability is similarly related to PA in other age groups besides adults, because neighborhood physical environmental interventions affect all inhabitants of a neighborhood, and not only adults. In Belgium, the relation between objectively measured walkability and PA has been investigated in adults (20–65 yr), adolescents (13–15 yr), and older adults (>65 yr). Also the moderating effect of neighborhood socio-economic status (SES) in the relation between walkability and PA was investigated in these age groups. Investigating this moderating effect is important, as the known health disparities across socio-economic groups [12] need to be reduced. Therefore, it is essential to investigate whether walkability is positively related to children’s physical activity in low SES neighborhoods. If a positive relation can be found, this can be promising for potential future interventions with a focus on children from low SES neighborhoods.

In Belgian adults, objectively measured walkability was positively associated with accelerometer-based MVPA, transport-related walking and cycling and recreational walking. No interactions between walkability and neighborhood SES were found [9]. In Belgian adolescents, objectively measured neighborhood walkability was positively associated with accelerometer-MVPA and the average activity level (counts/min), but only in low SES neighborhoods. In Belgian older adults, objectively measured walkability was positively related to transport-related walking. Furthermore, walkability was positively related to accelerometer-based MVPA [13]. As these studies investigated these relationships in the same neighborhoods, results in different age groups are comparable.

Based on these Belgian studies it can be concluded that objectively measured walkability is differently related to PA in different age groups [9,14]; but until now, no results on these associations are available in children. As physical environmental interventions can affect large groups of people at the same time, there is a strong need to investigate the association between objectively measured walkability and children’s PA before intervening in the environment.

Some international studies already investigated the relation between objectively measured walkability and children’s active transportation. Positive relations have been reported between school walkability and children’s active transportation to school in Denmark [15], Australia [16,17] and Canada [18]. One study investigated home neighborhood walkability in relation to active transportation to school in 5–18 year-old children in the USA [19]. In that study, walkability was positively related to children’s active transportation to school, only in high SES neighborhoods [19]. No European studies investigating the relation between objectively measured neighborhood walkability and children’s PA could be located in the literature; so there is a need for European studies investigating this relation.

The aim of this study was to investigate the association between objective neighborhood walkability and PA, and the possible moderating effects of neighborhood SES in this relation in Belgian children. This study is unique as the results will enable us to compare the differences in the relation between walkability and PA across different age groups in the same city.

## Methods

### Procedure

Data were collected between December 2011 and May 2013 as part of the Belgian Environmental Physical Activity Study in children (BEPAS-child). Principals (n = 46) from primary schools in Ghent (237000 inhabitants, 15685 km^2^) were asked to participate in the study. In total, 18 agreed and gave written informed consent (response rate = 34.6%). Children and their parents from fourth, fifth and sixth grade were informed (n = 994) about the study and 606 parents gave written informed consent (response rate = 61.0%). Of these 606 children, 112 children were excluded as no objectively measured walkability data were available (69 children did not live in Ghent and parents of 43 children did not fill out children’s home address in the questionnaire).

Children were asked to wear an accelerometer for 7 consecutive days, to fill out a questionnaire at school and one of the parents was asked to fill out a questionnaire together with his/her child. The Ethics Committee of the Ghent University Hospital approved the study.

### Measurements

#### Demographic variables

Sex was derived from the children’s questionnaire that was filled out in the classroom. Children’s age was derived from the parental questionnaire. Educational attainment was used as a proxy for family SES, as educational attainment is easy to measure and is fairly stable beyond early adulthood. Furthermore, higher levels of education are usually associated with better jobs, housing, neighborhoods, working conditions and higher incomes [20]. Parents were asked to report their own and their partner’s level of education (response options: primary school education, vocational secondary education, technical secondary education, general secondary education or art secondary education, college education or university education). Educational attainment was used as a proxy for family SES. Families were classified as high SES-families if the educational level of at least one parent was of a college or university education level; otherwise they were classified as low SES families.

#### Physical activity

Children’s self-reported PA was measured with the Flemish Physical Activity Questionnaire (FPAQ). Parents were asked to fill out the questionnaire at home together with their children and to report their child’s PA levels in a usual week. This questionnaire has been shown to be a reliable and reasonably valid instrument to assess different dimensions of PA in children, especially when completed with parental assistance [21]. The test–retest ICC’s ranged from 0.74 to 0.93, with exception from ICC = 0.26 for active transportation during leisure time. Concurrent validity ranged from r = 0.27 to r = 0.44 [21]. The number of minutes per day of walking for transport during leisure time, cycling for transport during leisure time, active transportation to school and sports during leisure time were derived from the questionnaire. An outline of the questionnaire is added as in Additional file 1.

Objective PA was determined by accelerometers. Children wore an Actigraph™ GT1M, GT3X or GT3X + accelerometer (15 s epoch) during waking hours for 7 consecutive days. Strong agreement was found between these three activity monitors for measuring MVPA in children [22], making it acceptable to use different models within a given study. The accelerometer was worn on the right hip. Accelerometer data were screened, cleaned and scored using data-reduction software MeterPlus 4.2. Periods of 20 minutes of consecutive zeros or more were removed and defined as non-wear time. Non-wear time activity diaries were provided to register activities for which the accelerometer was removed (e.g. bathing, swimming) and were used to replace the consecutive number of zeros by the corrected number of minutes MVPA [23]. MVPA was calculated using the cutpoints of Evenson [24]. These cutpoints were recommended in a comparative validity study of accelerometer cutpoints [25]. Children were included in the study if they had at least 2 weekdays with minimum 10 h wearing time or 1 weekend day with minimum 8 h wearing time [26].

#### Neighborhood variables

##### Neighborhood SES

Ghent consists of 201 statistical sectors, these are the smallest administrative entities for which statistical data, are available. Children included in the present study lived in 109 different statistical sectors. Median annual household income data (National Institute of Statistics–Belgium, 2008) were used to determine neighborhood SES of the different statistical sectors. Neighborhoods were characterized as low SES (income < €22,359) or high SES (income ≥ €22,359) based on the median.

##### Walkability

Objective neighborhood walkability of all statistical sectors was calculated using a geographical information system database. Geographical data were obtained from the Service for Environmental Planning in Ghent.

Residential density, intersection density and land use mix diversity were determined and z-scores were calculated. Walkability was calculated as follows: walkability = (2*z-connectivity) + (z-residential density) + (z-land use mix). This formula is an adapted version of the formula of Frank and colleagues [27]. Because no data of ‘retail floor area’ were available, this was omitted from the original formula. Residential density was calculated using the ratio of residential units to the land area devoted to residential use in each neighborhood. Connectivity in each neighborhood was represented by the ratio of the number of intersections (3 or more streets) to the land area. Land use mix indicated the degree of diversity of land use types in each neighborhood. Five land use types were considered: residential, retail, office, institutional, and recreational. Neighborhoods (i.e. statistical sectors) were characterized as low walkable or high walkable, based on the median.

### Analyses

Descriptive characteristics of the sample were analyzed using SPSS20. PA variables were logarithmically transformed (log10) to improve normality. Linear regression analyses were conducted in MLwiN2.25. Multilevel modeling was used to take into account clustering of children within classes within schools; and schools, classes and neighborhoods were treated as cross-classified. Model parameter estimates were obtained via Markov Chain Monte Carlo (MCMC) procedures applying an orthogonal parameterization [28].

To test the interaction between neighborhood walkability and neighborhood SES, the cross-product term of both variables was included in the models. When a significant interaction was found; separate models were fitted for high and low SES neighborhoods. All analyses were controlled for family SES, sex, age of the child and accelerometer wear time if relevant. P-values ≤ 0.05 were considered as significant with exception for the interaction terms where it was set at p ≤ 0.1. Higher significance levels are used for interaction terms as they have less power [29]. 95% confidence intervals (=CI) were reported.

## Results

### Descriptive results

Of the participating children; 45.1% were boys and 37.1% had a low family SES. The mean age of the sample was 10.9 ± 0.9 years. Children walked on average 6.6 ± 11.6 mins/day for transportation, cycled 4.7 ± 9.1 mins/day for transportation, engaged in active transportation to school for 5.1 ± 7.7 mins/day and engaged in sports for 20.2 ± 20.2 mins/day. Children engaged on average for 60.2 ± 23.5 mins/weekday and 50.0 ± 30.6 mins/weekend day in objective MVPA. In the sample, 59.4% of the children with low neighborhood SES and 79.3% of the children with high neighborhood SES were member of a sports club. PA levels by neighborhood walkability and SES are presented in Table 1.

**Table 1:** **Descriptive characteristics of the sample by neighborhood walkability and SES**

**Variable**		**High neighborhood walkability**		**Low neighborhood walkability**	
	**TOTAL GROUP**	**Low SES n = 197**	**High SES n = 48**	**Low SES n = 48**	**High SES n = 201**
Age (years ± SD)	10.09 ± 0.9	11.01 ± 0.9	11.01 ± 0.9	10.96 ± 1.0	10.81 ± 0.9
Sex (% boys)	45.1	46.7	45.8	50.0	42.3
Family SES (% low SES)	37.1	51.1	20.8	42.6	26.4
**FPAQ**	**mean ± SD mins/day**	**mean ± SD mins/day**	**mean ± SD mins/day**	**mean ± SD mins/day**	**mean ± SD mins/day**
Walking during leisure time	6.6 ± 11.6	11.3 ± 14.0	4.6 ± 9.3	3.5 ± 7.9	3.3 ± 8.1
Cycling during leisure time	4.7 ± 9.1	5.0 ± 9.8	5.3 ± 10.2	3.9 ± 5.5	4.6 ± 8.9
Active transportation to school	5.1 ± 7.7	5.7 ± 8.6	4.8 ± 8.8	3.7 ± 6.3	4.8 ± 7.2
Sports during leisure time	20.2 ± 20.2	16.2 ± 19.1	22.1 ± 18.2	25.4 ± 24.4	22.4 ± 20.1
Accelerometer					
MVPA on a weekday	60.2 ± 23.5	56.0 ± 23.2	64.2 ± 24.2	63.6 ± 19.7	60.3 ± 24.3
MVPA on a weekend day	50.0 ± 30.6	47.1 ± 26.8	54.8 ± 32.8	41.5 ± 24.1	53.6 ± 33.9

SD = standard deviation.

SES = socio- economic status.

MVPA = moderate- to vigorous-intensity physical activity.

FPAQ = Flemish physical activity questionnaire.

### Associations between neighborhood walkability, neighborhood SES and PA

**Figure 1:**
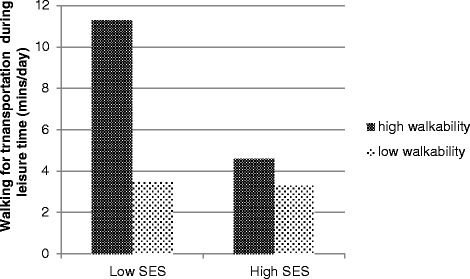
**Moderating effect of neighborhood SES in the relation between neighborhood walkability and walking for transportation.**

**Figure 2:**
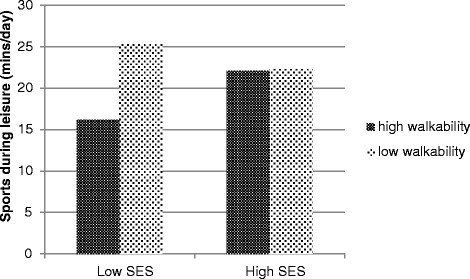
**Moderating effect of neighborhood SES in the relation between neighborhood walkability and sports during leisure time.**

A significant interaction between neighborhood walkability and neighborhood SES was found in relation to walking for transportation during leisure time (β = −0.251 ± 0.126; 95% CI = −0.498, −0.004) (Table 2). In low SES neighborhoods, walkability was positively related to walking for transportation during leisure time (β = 0.381 ± 0.124; 95% CI = 0.138, 0.624) whereas in high SES neighborhoods, walkability was unrelated to walking for transportation during leisure time (β = 0.130 ± 0.087; 95% CI = −0.582, 0.562) (Figure 1). In the relation between walkability and sports during leisure time, a significant moderating effect of neighborhood SES was found (β = 0.297 ± 0.152; 95% CI = −0.001, 0.595) (Figure 2). In low SES neighborhoods, walkability was negatively related to sports during leisure time (β = −0.245 ± 0.121; 95% CI = −0.482, −0.008); whereas in high SES neighborhoods walkability was unrelated to sports during leisure time (β = 0.004 ± 0.113; 95% CI = −0.217 and 0.225). No other interaction effects were found (Table 2).

**Table 2:** **Associations between SES, walkability and physical activity**

	**Walking for transportation during leisure time** ^**a**^	**Cycling for transportation during leisure time** ^**a**^	**Active transportation to school** ^**a**^	**Sports during leisure time** ^**a**^	**MVPA on a weekday** ^**a,b**^	**MVPA on a weekend day** ^**a,b**^
	**n = 473 β ± SE**	**n = 474 β ± SE**	**n = 472 β ± SE**	**n = 474 β ± SE**	**n = 432 β ± SE**	**n = 397 β ± SE**
Sex (ref = boy)	0.019 ± 0.044	−0.067 ± 0.046	0.036 ± 0.048	−0.213 ± 0.055**	−0.147 ± 0.015**	−0.076 ± 0.029*
Age	0.004 ± 0.028	0.038 ± 0.027	0.048 ± 0.030	0.011 ± 0.033	−0.019 ± 0.010	−0.027 ± 0.018
Family SES (ref = low)	−0.189 ± 0.051**	0.031 ± 0.051	0.001 ± 0.055	0.310 ± 0.062**	0.031 ± 0.017	0.071 ± 0.033*
Neighborhood walkability (ref = low)	0.363 ± 0.096**	−0.040 ± 0.096	0.009 ± 0.107	−0.267 ± 0.113*	−0.036 ± 0.034	0.103 ± 0.064
Neighborhood SES (ref = low)	−0.051 ± 0.090	−0.016 ± 0.094	0.059 ± 0.097	−0.017 ± 0.109	−0.037 ± 0.031	0.116 ± 0.059*
Neighborhood walkability * neighborhood SES	−0.251 ± 0.126*	0.035 ± 0.131	0.070 ± 0.142	0.297 ± 0.152(*)	0.051 ± 0.043	−0.068 ± 0.082

(*) 0.05 < p < 0.10, *p < 0.05, **p < 0.001.

^a^ = logarithmically transformed.

^b^ = controlled for accelerometer wear time.

n = number of children in the analytical sample.

β = multilevel linear regression coefficient.

SE = standard error.

Neighborhood walkability was unrelated to weekend day accelerometer-MVPA (β = 0.103 ± 0.064; 95% CI = −0.022 and 0.228), and neighborhood SES was positively related to weekend day accelerometer-MVPA (β = 0.116 ± 0.059; 95% CI = 0.001 and 0.232). Neighborhood walkability and neighborhood SES were unrelated to cycling for transportation during leisure time, active transportation to school and weekday accelerometer-based MVPA.

### Associations of socio-demographic covariates with PA

Girls engaged less in sports during leisure time (β = −0.213 ± 0.055; 95% CI = −0.321 and −0.105) and did less weekday MVPA (β = −0.147 ± 0.015; 95% CI = −0.176 and −0.118) and weekend MVPA (β = −0.076 ± 0.029; 95% CI = −0.133 and −0.019) compared to boys. Family SES was negatively related to walking for transportation during leisure time (β = −0.189 ± 0.051; 95% CI = −0.289 and −0.089), and positively related to sports during leisure time (β = 0.310 ± 0.062; 95% CI = 0.188 and 0.432) and MVPA during weekend days (β = 0.071 ± 0.033; 95% CI = 0.006 and 0.136) (Table 2).

## Discussion

The aim of this study was to investigate the association between objectively measured neighborhood walkability and children’s PA and the possible moderating effects of neighborhood SES in this association. As the moderating effect of neighborhood SES affects the direct relation between neighborhood walkability and children’s physical activity, the moderating effects will be discussed first.

Two significant moderating effects of neighborhood SES were found. The positive relation between walkability and walking for transportation during leisure was only present in low SES neighborhoods. It is assumed that children living in high SES neighborhoods have more access to motorized transport, and are therefore less dependent on the walkability of their neighborhood; whereas children from low SES neighborhoods may have less access to motorized transport and these children walk more for transportation during leisure time in a high walkable neighborhood, when a lot of destinations are nearby. However, this moderating effect was not found for active transportation to school. It is possible that both children from high and low SES neighborhoods go to school actively, as the prevalence of active commuting in Belgian children is high (59.3% actively commutes to school) [30], possibly because most of children live close to their school [30]. Therefore, children’s active transportation to school is probably independent of the neighborhood walkability and SES in Belgium.

Neighborhood SES also moderated the relation between walkability and sports during leisure time. In low SES neighborhoods, children engaged more in sports in low walkable neighborhoods and less in high walkable neighborhoods; whereas in high SES neighborhoods, walkability was unrelated to sports. It has been shown previously that SES was positively associated with sports club membership [31]. As children from high SES neighborhoods are more frequently a member of a sports club; the characteristics of their neighborhood are probably less important in order to be active. Because children from low SES neighborhoods do not always have the opportunity to be member of a sports club, due to high costs [32] and lack of parental support, they probably engage more in unorganized forms of PA such as active street play (e.g. playing street soccer,..). In an Australian study, safety, living in a dead-end street and public open spaces were positively associated with children’s play [33]. Also in the USA [34] and Canada [35] children were more active in their neighborhood when low street connectivity was perceived (e.g. more dead-end streets). As these environmental factors are mainly characteristics of a low walkable neighborhood, this may explain the negative relation between walkability and sports during leisure time in low SES neighborhoods.

The fact that children from high SES are more frequently member of a sports club [31], may also explain the positive relation between neighborhood SES and children’s weekend MVPA and between family SES and sports during leisure time. However this relation was not found for weekday MVPA. As children’s weekday MVPA is highly dependent on the MVPA of children during the school day, it could be expected that children’s SES or walkability was unrelated to their weekday MVPA. Furthermore, no effects of walkability or neighborhood SES were found on children’s MVPA. These results show that environmental factors are differently related to specific domains of PA, which argues for investigating the relation between the environment and PA for different domains of PA, rather than investigating this in relation to overall MVPA or total PA [36].

Different relations between the environment and PA were found across different age groups in the same city [9,13,14]. But similar as in Belgian adolescents in the same city, we found a stronger relation between neighborhood walkability and PA in low SES neighborhoods [14]. However, there is no univocal relation between neighborhood walkability and children’s PA. Living in a high walkable neighborhood can be beneficial for walking for transportation during leisure time, but is negatively associated with sports during leisure time in low-SES neighborhoods. Therefore, based on the present findings the positive and promising results from the adult studies cannot be generalized to children [8-10]. This raises the question whether the walkability index, should be changed into a “playability” or “movability” index in order to be relevant to explain children’s PA, as it is possible that other environmental variables (e.g. open spaces and dead end streets), are more important. This implies that physical environmental interventions targeted to increase PA in adults may have opposite effects in children, so attention should be paid when intervening in the physical environment. Children must have the opportunity to play outside and to be active in their neighborhood, especially in low SES and high walkable neighborhoods. Therefore, future interventions should focus on economically disadvantaged subgroups. This may be done by providing play space for children from low SES neighborhoods, e.g. by the provision of sport fields or play streets.

Also the relation between environmental parental perceptions and children’s PA needs to be investigated. As parents are seen as the decision makers for their children [37], it is possible that parental environmental perceptions are more strongly related to children’s PA.

A strength of this study is the use of objectively measured walkability, as objective measures have less measurement error compared to perceptions [38]. Furthermore, the relation between walkability and PA was measured objectively and subjectively. The cross-sectional character of the study is a limitation, as no causal relationships can be examined. Second, statistical sectors were used to define neighborhoods. It is possible that the investigated neighborhood was not the neighborhood that children and parents would define as ‘their own neighborhood’. Also the low response rate of the school principals is a limitation of this study. Furthermore, data were collected in one city, which may limit the generalizability of the findings. Also, children were recruited in schools instead of neighborhoods with varying walkability and SES levels. This led to the inclusion of children living mostly in high SES–low walkable or low SES–high walkable neighborhoods; children living in low SES–low walkable and high SES–high walkable neighborhoods were underrepresented. Besides, it needs to be acknowledged that it is very difficult to point out the exact relation between physical environmental factors and physical activity, because of the strong interaction between environmental, social and individual factors. Therefore, in future research, the moderating effect of other factors (e.g. family type, number of siblings) in the relation between walkability and children’s physical activity should be investigated.

## Conclusions

No univocal relation between walkability and PA was found in children. In low SES neighborhoods, children walked more for transportation during leisure time and engaged in less sports during leisure time in high walkable neighborhoods compared to low walkable neighborhoods. In high SES neighborhoods, walkability was unrelated to children’s PA. Results from international adult studies cannot be generalized to children.

## Additional file

Additional file 1:
**Outline of the FPAQ questionnaire.**

## Abbreviations

MVPA: Moderate- to vigorous-intensity physical activity
PA: Physical activity
