# Supplementary material for: The association between objective walkability, neighborhood socio-economic status, and physical activity in Belgian children
Source: Int J Behav Nutr Phys Act. 2014 Aug 23;11:104. doi: 10.1186/s12966-014-0104-1 (PMC4243938; doi:10.1186/s12966-014-0104-1)
Supplement: Additional file 1: — Outline of the FPAQ questionnaire. [file 12966_2014_104_MOESM1_ESM.docx]

**Additional file 1.** **Outline of the FPAQ questionnaire**

By answering the following questions, face a ‘usual’ week (7 days)

1. How does your child usually go to school?

O on foot

O by bike

O by car

O by bus, train or tram

1. The home-school trip, with the transport mode reported in question 1, lasted:

O 1-5 minutes

O 6-10 minutes

O 11-15 minutes

O 16-20 minutes

O more than 20 minutes

1. Does your child usually walk to destinations on weekdays?

(e.g. to the sports club, to the bakery, to shops,..)

=> Do not include active transportation to school and walking as a sport.

O yes O no

If yes; how many minutes does your child usually walk per weekday?

O 1 - 10 minutes O 40 - 50 minutes

O 10 - 20 minutes O 50 - 60 minutes

O 20 - 30 minutes O 1 hour – 1 hour 10 minutes

O 30 - 40 minutes O more than 1 hour and 10 minutes

1. Does your child usually walk to destinations on weekend days?

(e.g. to the sports club, to the bakery, to shops,..)

=> Do not include walking as a sport.

O yes O no

If yes; how many minutes does your child usually walk per weekend day?

O 1 - 10 minutes O 40 - 50 minutes

O 10 - 20 minutes O 50 - 60 minutes

O 20 - 30 minutes O 1 hour – 1 hour 10 minutes

O 30 - 40 minutes O more than 1 hour and 10 minutes

1. Does your child usually cycle to destinations on weekdays?

(e.g. to the sports club, to the bakery, to shops,..)

=>Do not include active transportation to school and cycling as a sport.

O yes O no

If yes; how many minutes does your child usually cycle per weekday?

O 1 - 10 minutes O 40 - 50 minutes

O 10 - 20 minutes O 50 - 60 minutes

O 20 - 30 minutes O 1 hour – 1 hour 10 minutes

O 30 - 40 minutes O more than 1 hour and 10 minutes

1. Does your child usually cycle to destinations on weekend days?

(e.g. to the sports club, to the bakery, to shops,..)

=>Do not include cycling as a sport.

O yes O no

If yes; how many minutes does your child usually cycle per weekend day?

O 1 - 10 minutes O 40 - 50 minutes

O 10 - 20 minutes O 50 - 60 minutes

O 20 - 30 minutes O 1 hour – 1 hour 10 minutes

O 30 - 40 minutes O more than 1 hour and 10 minutes

1. Give the three main sports your child practices most in leisure time.

(note: physical education and school sports do not count for sports in leisure time)

My first sport

(similar for second and third)

O I don’t practice a sport in leisure time

O My first sport is: …………………….

Does your child practice this sport in a sports club? O yes O no

| How often do you practice this sport?  (indicate only 1 option) | How much time do you spend on this sport per week? |
| --- | --- |
| O Now and then but not every week  O one time per week  O two times per week  O three times per week  O four times per week  O five times per week  O six times per week  O seven times per week  O more than seven times a week | .......... hours ……… minutes per week |
